# The Type 2 Deiodinase Thr92Ala Polymorphism Is Associated with Higher Body Mass Index and Fasting Glucose Levels: A Systematic Review and Meta-Analysis

**DOI:** 10.1155/2021/9914009

**Published:** 2021-10-07

**Authors:** Xichang Wang, Kan Chen, Chenyu Zhang, Haoyu Wang, Jiashu Li, Chuyuan Wang, Weiping Teng, Zhongyan Shan, Yaxin Lai

**Affiliations:** Department of Endocrinology and Metabolism and the Institute of Endocrinology, The NHC Key Laboratory of Diagnosis and Treatment of Thyroid Diseases, The First Hospital of China Medical University, Shenyang 110001, China

## Abstract

**Background:**

Type 2 deiodinase (Dio2) is a selenoenzyme that is mainly expressed in the endoplasmic reticulum of the central nervous system, brown adipose tissue, and placenta and is responsible for outer ring deiodination of thyroxine (T4) to form biologically active triiodothyronine (T3). The Thr92Ala polymorphism of Dio2 has been found to be a potential risk factor for various diseases beyond the hypothalamus-pituitary-thyroid (HPT) axis.

**Methods:**

We searched the relevant studies in the PubMed, Embase, and Cochrane Library databases and Google Scholar. A systematic review and meta-analysis of studies on the Thr92Ala polymorphism and metabolic parameters beyond the HPT axis (e.g., BMI, fasting glycemic traits, plasma lipid levels, and hypertension risk) were performed.

**Results:**

Six eligible studies that analyzed the relationship between the Thr92Ala polymorphism and metabolic parameters beyond the thyroid were identified. All selected studies excluded patients with thyroid dysfunction, and diabetic patients were also excluded when fasting glucose and fasting insulin levels were meta-analyzed. The Thr92Ala polymorphism was found to be a significant risk factor for higher BMI (Std. mean difference 0.31 (0.01, 0.60), *p* = 0.04) and higher fasting glucose levels (Std. mean difference 1.18 (0.05, 2.31), *p* = 0.04). However, fasting insulin levels, plasma lipid levels, and hypertension risk showed a nonsignificant association with the Thr92Ala polymorphism.

**Conclusion:**

Compared with euthyroid noncarriers (Thr/Thr), euthyroid Ala92-Dio2 carriers showed increased BMI levels, and Ala92-Dio2 carriers also had higher fasting plasma glucose levels than matched euthyroid nondiabetic noncarriers.

## 1. Introduction

Thyroid hormones are indispensable for maintaining the normal physiological function of various systems during the lifetime [1] and mainly consist of thyroxine (T4) and triiodothyronine (T3). Interestingly, approximately 80% of T3 in humans is not secreted by the thyroid itself but is formed by the deiodination reaction of T4. Moreover, T3 is the biologically active form of thyroid hormone, and its affinity with receptors is ten times higher than that of T4 [2]. The above evidence shows that deiodinase plays an important role in maintaining the ratio of T3/T4 and the metabolic function in humans, both generally and in local sites. Deiodinase is composed of three subtypes, namely, type 1 (Dio1), type 2 (Dio2), and type 3 (Dio3), and of these, Dio1 and Dio2 are mainly responsible for catalyzing the outer ring (5′) deiodination reaction of T4 to produce sufficient T3. The different intracellular sites of Dio1 and Dio2 determine their different physiological functions. Dio1 is located in the plasma membrane and is responsible for producing circulating T3, whereas Dio2 is located in endoplasmic reticulum (ER) vesicles and catalyzes the local production of T3 in specific cells [3]. Therefore, for tissues with high expression of Dio2, such as the central nervous system, brown adipose tissue, placenta, and skeletal muscle, the local metabolic function depends to a large extent on the activity of Dio2.

The Thr92Ala polymorphism of Dio2 means a Thr92-to-Ala substitution caused by a SNP (rs225014) that is prevalent in 12%-36% of the population [4] and is currently the most widely studied polymorphic site of Dio2. Not surprisingly, the Thr92-to-Ala substitution reduces the catalytic efficiency of Dio2 and induces localized hypothyroidism [5]. It has been demonstrated that hypothyroid carriers of Ala92 gain little benefit from levothyroxine monotherapy, even if their thyroid-stimulating hormone (TSH) levels decrease to normal [6, 7]. Therefore, the Thr92-to-Ala substitution might predict a need for a higher levothyroxine dose for hypothyroidism patients [8]. On the other hand, studies have also found that Ala92 might play a protective role for patients with Graves' disease, and that the frequency of disease development, the severity of clinical manifestations, and the risk of remission were much lower for Ala92 carriers than for noncarriers [9, 10].

In addition to acting as a potential risk factor for abnormal thyroid function and abnormal therapeutic effects of thyroid medications, Thr92Ala might also correlate with several diseases beyond the hypothalamus-pituitary-thyroid (HPT) axis. It has been hypothesized that Thr92Ala is significantly associated with metabolic disorders such as type 2 diabetes [11], insulin resistance [12], hypertension [13], and osteoporosis [14]. However, the endocrinology community has not yet reached a consensus on the impact of the Thr92Ala polymorphism on various metabolic disorders. In a cross-sectional study in Denmark, 7,342 subjects were genotyped, and no significant association was detected between Thr92Ala and type 2 diabetes, insulin resistance, and obesity [15]. The Amish Family Diabetes Study verified that there was no significant difference in fasting glucose or fasting insulin levels between different genotypes of Thr92Ala; interestingly, the Ala92 allele was found to be associated with increased insulin sensitivity with a glucose tolerance test [16]. Wouters et al. compared several clinical characteristics between different genotypes and found no significant difference in blood pressure, plasma glucose traits, plasma lipid levels, or BMI [17]. Therefore, the association between Thr92Ala and various metabolic parameters beyond the thyroid requires further integration and analysis.

The purpose of the present study was to explore the association between Thr92Ala and metabolic phenotypes such as BMI, fasting glycemic traits, plasma lipid levels, and hypertension risk to provide more evidence to support the association between the polymorphism or activity of Dio2 and metabolic disorders beyond the HPT axis.

## 2. Materials and Methods

### 2.1. The Search Strategy for Studies

Two investigators independently conducted the literature search in the PubMed, Embase, and Cochrane Library databases and Google Scholar in sequence until 8 December 2020. The keywords “Thr92Ala,” “rs225014,” “Dio2 A/G,” and “deiodinase polymorphism” were used. Moreover, the references of all initially included articles, relevant reviews, and meta-analyses were also manually screened by full-text examination. We used PubMed as the main retrieval tool, supplemented by other tools mentioned above and the reference lists of relevant papers. The searching formula on PubMed was as follows: (((Thr92Ala[Title/Abstract]) OR (rs225014[Title/Abstract])) OR (Dio2 A/G[Title/Abstract])) OR (deiodinase polymorphism[Title/Abstract]).

### 2.2. Inclusion and Exclusion Criteria for Articles

**Figure 1:**
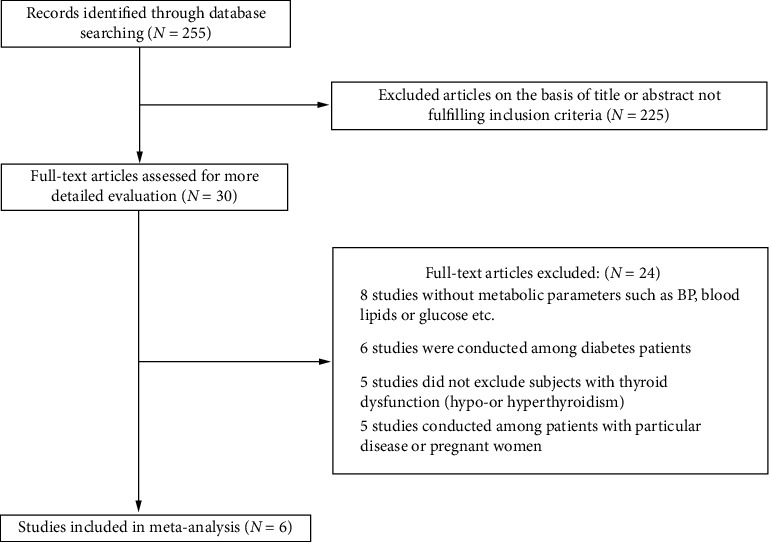
Flow diagram for the literature search.

The overall process of searching the literature is shown in Figure 1. Articles were selected if they met all of the following criteria: (1) observational study regarding Dio2 polymorphism; (2) metabolic indicators assessed, with one or more of the following: BMI, fasting glucose level, fasting insulin level, total cholesterol level, total triglyceride level, high-density lipoprotein cholesterol level, low-density lipoprotein cholesterol level, or hypertension prevalence; (3) subjects with thyroid dysfunction (hypo- or hyperthyroidism) excluded; and (4) for studies analyzing fasting glycemic traits, subjects with a personal history of diabetes were excluded.

Articles were excluded if they met one of the following criteria: (1) studies that did not detect Thr92Ala (rs225014) specifically, (2) studies on pregnant women, and (3) studies conducted among patients with particular diseases (such as diabetes).

### 2.3. Data Extraction

As shown in Table 1, the relevant information from all selected studies was extracted and recorded, including the first author, publication year, age and sex distribution, sample size of each genotype, allele frequency of alanine, and metabolic parameters. The specific values of metabolic phenotypes (mean ± standard deviation for continuous variables and case numbers for dichotomous variables) were also recorded elsewhere.

### 2.4. Quality Assessment

Quality assessment was conducted by two persons in parallel with the Newcastle-Ottawa scale (NOS) for nonrandomized controlled trials (RCTs) [18]. Disagreement was resolved by a third person if different scores were given. The maximum and minimum scores were nine and zero, respectively. Studies with five stars or more were ultimately included.

### 2.5. Statistical Analysis

Data from the final included studies were retrieved and annotated. The inverse variance method and Mantel-Haenszel method were applied when analyzing continuous variables and dichotomous variables, respectively. For continuous variables such as BMI and fasting glucose levels, the differences between different genotypes are presented in the form of standardized mean differences (SMDs). Moreover, dichotomous variables such as the hypertension prevalence of each genotype were also displayed, and the corresponding odds ratio (OR) was meta-analyzed.

The chi-squared-based *Q* test and *I*^2^ test were applied to estimate the heterogeneity of the included studies, and 75%, 50%, and 25% were identified as high, moderate, and low levels, respectively. A random-effects model was selected if the included studies showed moderate or high heterogeneity; otherwise, a fixed-effects model was selected. Sensitivity analysis was performed by sequential removal of each study. Meta-analysis was conducted using *Review Manager (RevMan) [Computer program]. Version 5.4.1, The Cochrane Collaboration, 2020.*

## 3. Results

### 3.1. Characteristics of Included Studies

As shown in Figure 1, 255 articles were initially found by database searching. Thirty articles remained after examining the titles and abstracts. Then, we assessed these articles in more detail by careful full-text examination, and six articles (seven studies) were ultimately included [13, 19–23], of which van der Deure et al. analyzed the impact of Thr92Ala polymorphism on hypertension risk in two separate populations [23]. Although Kang et al. compared the differences in fasting glucose and fasting insulin levels between different genotypes, we did not include the above data because diabetes patients were not excluded. The detailed demographic information of each study is displayed in Table 1.

### 3.2. Association between the Thr92Ala Polymorphism and BMI

**Figure 2:**
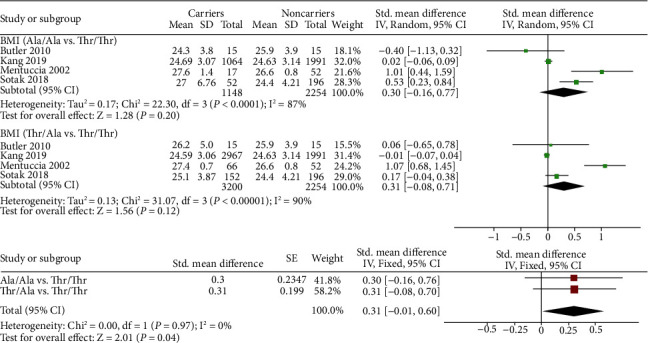
Pairwise comparison and standardized mean difference integration of the body mass index of different genotypes.

Four studies were included in the meta-analysis between Thr92Ala and BMI [19–22]. In total, 2,254 subjects with Thr/Thr, 3200 subjects with Thr/Ala, and 1148 subjects with Ala/Ala were included. We compared the differences in BMI between noncarrier subjects (Thr/Thr) and Ala carrier subjects (Thr/Ala or Ala/Ala). As shown in Figure 2, the results showed that although there was no significant difference in SMD within either subgroup (SMD 0.30 (-0.16, 0.77) for Ala/Ala vs. Thr/Thr and SMD 0.31 (-0.08, 0.71) for Thr/Ala vs. Thr/Thr), the overall BMI of noncarrier subjects was significantly lower than that of carrier subjects (SMD 0.31 (0.01, 0.60), *p* = 0.04).

### 3.3. Association between the Thr92Ala Polymorphism and Glycemic Traits

**Figure 3:**
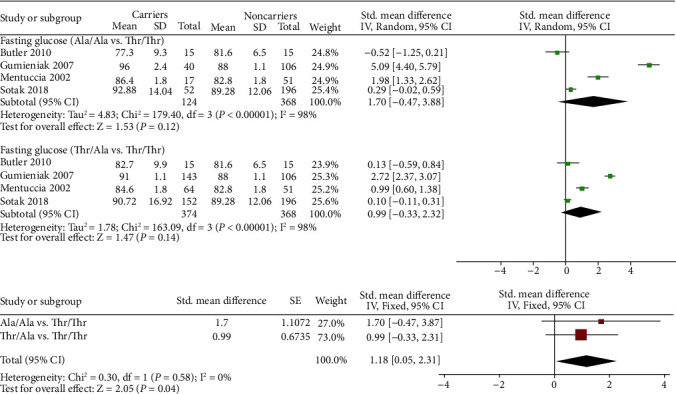
Pairwise comparison and standardized mean difference integration of the fasting glucose levels of different genotypes.

**Figure 4:**
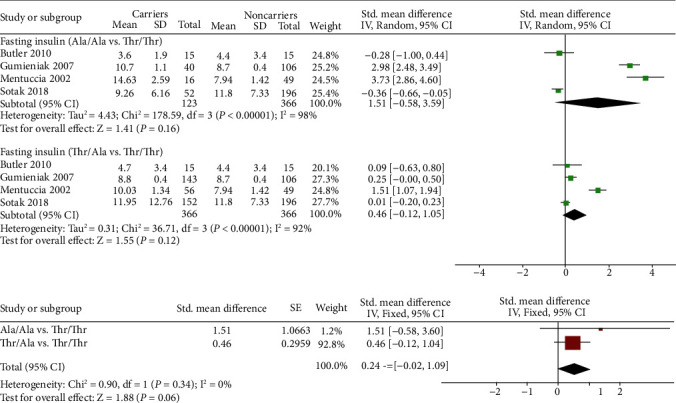
Pairwise comparison and standardized mean difference integration of the fasting insulin levels of different genotypes.

As shown in Figures 3 and 4, four [13, 19, 21, 22] studies were included when fasting glucose or fasting insulin levels were analyzed.

A total of 368 subjects with Thr/Thr, 374 subjects with Thr/Ala, and 124 subjects with Ala/Ala were included when we compared the differences in fasting glucose levels. Similar to the previous BMI results, when we calculated the SMD of fasting glucose levels in pairs, no significant result was found within the two subgroups (SMD 1.70 (-0.47, 3.88) for Ala/Ala vs. Thr/Thr and SMD 0.99 (-0.33, 2.32) for Thr/Ala vs. Thr/Thr). However, the overall fasting glucose levels of carriers were significantly higher than those of noncarriers (SMD 1.18 (0.05, 2.31), *p* = 0.04) (Figure 3).

However, the results of fasting insulin levels were somewhat different from those of fasting glucose levels (Figure 4). There was no significant difference between 366 Thr/Thr subjects and 123 Ala/Ala subjects (SMD 1.51 (-0.58, 3.59)) or between 366 Thr/Thr subjects and 366 Thr/Ala subjects (SMD 0.46 (-0.12, 1.05)). Similarly, nonsignificant results were shown when we meta-analyzed the above two subgroups of SMD (0.54 (-0.02, 1.09), *p* = 0.06), indicating that the fasting insulin level of carrier subjects was comparable to that of noncarrier subjects.

### 3.4. Association between Thr92Ala Polymorphism and Plasma Lipid Levels

Two studies were included when the difference in plasma lipid levels between different genotypes was analyzed [19, 20]. As shown in Supplementary Figure 1 to Supplementary Figure 3, within the two subgroups or in general, the SMDs in total cholesterol (TC), total triglyceride (TG), or high-density lipoprotein cholesterol (HDL) levels all showed no significant differences (overall *p* value: *p* for TC = 0.25, *p* for TG = 0.91, and *p* for HDL = 0.60).

### 3.5. Association between the Thr92Ala Polymorphism and Hypertension Risk

Two articles (three studies) were meta-analyzed with regard to the impact of the Thr92Ala polymorphism on hypertension risk [13, 23]. We recalculated the overall hypertension prevalence in the two carrier subgroups and compared it with the prevalence in the noncarrier subgroup. As shown in Supplementary Figure 4, there was no significant difference in prevalence between the carriers and noncarriers (OR 1.14 (0.81, 1.61), *p* = 0.45).

## 4. Discussion

In the present meta-analysis, we found for the first time that the Dio2 Thr92Ala polymorphism could lead to a significant increase in BMI among euthyroid participants. Furthermore, for subjects without diabetes or thyroid dysfunction, the polymorphism could significantly induce elevated fasting glucose levels. However, serum fasting insulin levels, serum lipid levels, and hypertension risk showed no significant difference between the different genotypes.

As Dio2 is the key enzyme that determines the ratio of T3/T4 in local tissues, its activity is essential for maintaining the physiological metabolic function of specific sites, such as the central nervous system, brown adipose tissue, and placenta. Previous studies have confirmed that if the threonine at position 92 of Dio2 is replaced by alanine, its catalytic activity will be reduced [24]. This in turn affects the production of bioactive T3 at local sites. A recent animal experiment also found that the manifestations of hypothyroidism in mice carrying the Ala92-Dio2 polymorphism were obvious in distinct brain areas. Acting as a cargo protein in ER and Golgi complex vesicles, Ala92-Dio2 causes ER stress and induces the unfolded protein response (UPR), and the specific symptoms can be improved by agents eliminating ER stress or the administration of LT3 [24]. Therefore, not surprisingly, Thr92Ala was hypothesized to be significantly associated with local T3 production in humans, thereby significantly affecting neuropsychiatric and metabolic functions.

There are currently several studies on the associations between the Thr92Ala polymorphism and BMI or obesity. From a basic perspective, a decrease in Dio2 activity would decrease the production of Dio2-generated T3 in various organs, inhibiting energy utilization and probably inducing a higher BMI. However, positive conclusions have rarely been drawn in epidemiological studies. In a case-control study conducted by Heemstra et al., it was demonstrated that among patients with Hashimoto thyroiditis, mean BMI was significantly elevated with the increase in Ala92 frequency [25]. However, another case-control study with 362 obesity patients and 127 controls found that none of the representative SNPs in the Dio2 gene were associated with obesity or BMI, including Thr92Ala [26], which was further verified in three other studies among diabetes patients [12, 27, 28] and one among thyroidectomized patients [8]. Similarly, none of the studies included in our meta-analysis can confirm the exact association between the Thr92Ala polymorphism and BMI alone. However, the results after integration suggested that among individuals without thyroid dysfunction, subjects carrying Thr92Ala had a significantly higher BMI than Thr/Thr subjects. In the future, we need more similar epidemiologic studies to verify the exact association between the two.

Among a series of metabolic disorders beyond the HPT axis, studies on the Thr92Ala polymorphism and type 2 diabetes are currently the most common. It has been indicated that a reduction in T3 production in skeletal muscle or the liver might induce local hypothyroidism and inhibit glucose disposal. In addition, the decreased level of Dio2-generated T3 might also inhibit the transcription of GLUT4 in insulin-sensitive tissues, such as adipose tissue or skeletal muscle, which could lead to insulin resistance [29]. We found in the present meta-analysis that the occurrence of Ala92-Dio2 was significantly associated with higher fasting glucose levels for nondiabetic euthyroid subjects, while fasting insulin levels were comparable by pairwise comparison or after integration. Therefore, although Ala92-Dio2 carriers generally have higher fasting glucose levels, whether the Thr92Ala polymorphism is associated with insulin resistance or diabetes risk remains to be explored. In contrast to our findings, another small-scale study showed that Ala92 frequency was not associated with fasting or 2-hour glycemic traits, but the hyperinsulinemic euglycemic clamp test indicated a lower insulin sensitivity for Ala92 carriers [26]. In addition, Estivalet et al. and Canini et al. demonstrated that fasting insulin levels in diabetic Ala/Ala subjects were significantly higher than that in subjects with the other two genotypes, whereas the difference in fasting glucose levels was nonsignificant between the three genotypes; accordingly, diabetic Ala/Ala carriers were likely to have a more pronounced homeostasis model assessment (HOMA) index [12, 27]. Nevertheless, in another study conducted by Peeters et al., Ala92 carriers showed an increased trend of fasting glucose levels, fasting insulin levels, and HOMA index, but none of the above trends reached statistical significance [30]. In general, most relevant studies suggest that Ala92 carriers have worse glycemic control [31], an increased risk of type 2 diabetes [11], or decreased insulin sensitivity [12, 21, 26, 27]. However, there is still insufficient evidence supporting the particular association between the Thr92Ala polymorphism and fasting glucose levels, especially among nondiabetic euthyroid subjects, which requires more research for confirmation.

In the present meta-analysis, we did not conclude a significant association between the Thr92Ala polymorphism and plasma lipid levels. Since there was no previous study that specifically analyzed the relationship between the two, the selected studies did not exclude subjects who took lipid-lowering treatments or had a personal history of dyslipidemia, which might have a certain bias on the results. In addition to the studies included in the present meta-analysis [19, 20], we did not find significant evidence indicating a strong link between the Thr92Ala polymorphism and serum lipid levels [12, 15, 17, 27, 28]. Therefore, we speculate that Ala92 does not cause significant alterations in plasma lipid levels. Based on the above evidence, we need to restrict the screening criteria for participants in future studies to specifically evaluate the association between the Thr92Ala polymorphism and serum lipid levels among euthyroid subjects without dyslipidemia.

Regarding the association between the Thr92Ala polymorphism and blood pressure value or hypertension risk, previous studies have reported positive conclusions. Studies have found a larger proportion of Ala92 carriers among hypertensive subjects than among normotensive subjects, and the Thr92Ala polymorphism could be regarded as a significant risk factor for hypertension after adjusting for confounding factors, especially in Black populations [13]. This study, which reported a positive conclusion, was also included in the present meta-analysis. Moreover, an earlier study by the same group found that normotensive subjects with a family history of hypertension generally had higher TSH levels than healthy controls [32]. The difference in genetic background may explain this finding. However, apart from the abovementioned evidence, we have not found a definitive conclusion for the association between Thr92Ala and blood pressure value or hypertension in previous studies [12, 17, 23, 27, 33]. Although all of the selected studies in the meta-analysis excluded subjects recently taking antihypertensive medications, Thr92Ala showed no significant association with hypertension risk, either within the pairwise comparison or in general. Currently, there are few studies on the issue regarding Dio2 polymorphism and hypertension risk; thus, our findings should be considered preliminary and require confirmation.

Heterogeneity is one of the unavoidable problems in meta-analysis, and this study is no exception. It is worth noting that this meta-analysis covers six countries or regions, including Slovakia, the United States, and South Korea. Therefore, geographic and ethnic heterogeneity may have an impact on the conclusions. There is inherent heterogeneity in the genetic background of the subjects. In addition, the diagnostic criteria for thyroid diseases are also heterogeneous. Although all studies have excluded patients with thyroid disease, only Butler et al. [19], Mentuccia et al. [21], and van der Deure et al. [23] evaluated the thyroid disease based on serum biochemical indicators, and Butler et al. [19] even further excluded patients with positive thyroid autoantibodies. The reference intervals for TSH and FT4 are also different, and these factors may all contribute to the heterogeneity. Therefore, when analyzing several metabolic indicators (i.e., BMI, fasting glucose level, fasting insulin level, and hypertension), we chose a random-effects model to obtain a wider confidence interval and relatively reliable conclusions. However, the related research currently has a small sample size, and it is inappropriate to conduct subgroup analysis based on characteristics such as age or gender. Therefore, the conclusion of this meta-analysis needs to be confirmed by more large sample surveys.

The limitations of the present meta-analysis are as follows. First, the elevation of various metabolic parameters beyond the thyroid is actually determined by multiple factors, and the proportion of genetic factors is still unknown. Second, we did not consider the impact of lipid-lowering treatments or personal history of dyslipidemia when analyzing plasma lipid levels, which might bias the final conclusion. Third, as mentioned above, the relevant studies have a relatively small sample size, and the heterogeneities of ethnicity, excluding criteria for thyroid patients, and reference interval for TSH all might induce the bias. More research is needed since genetic association research should recruit a large population to ensure convincible conclusions.

## 5. Conclusions

To the authors' knowledge, the present meta-analysis is the first study that clearly demonstrated the significant positive association between the Thr92Ala polymorphism and BMI among euthyroid subjects. For nondiabetic euthyroid subjects, the Thr92Ala polymorphism could also induce a higher fasting glucose level. However, fasting insulin levels, serum lipid levels, and hypertension risk showed no significant association with the Thr92Ala polymorphism.

## Figures and Tables

**Table 1:** Characteristics of the selected studies.

Author	Published year	Age composition	Sex composition (%female)	Number of Thr/Thr	Number of Thr/Ala	Number of Ala/Ala	Ala-allele frequency (%)	Metabolic parameters
Mentuccia	2002	42.3 ± 16.7	100.00%	52	66	17	37.04%	BMI, fasting glucose, fasting insulin
Gumieniak	2007	18-65	43.60%	143	175	54	38.04%	Hypertension prevalence, fasting glucose, fasting insulin
van der Deure WM (a)	2009	68.84 ± 7.57	60.66%	521	634	169	36.71%	Hypertension prevalence
van der Deure WM (b)	2009	72.04 ± 7.33	50.65%	376	459	135	37.58%	Hypertension prevalence
Butler	2010	18-65	60.00%	15	15	15	50.00%	BMI, fasting glucose, fasting insulin, TC, TG, HDL
Sotak	2018	20-92	63.00%	196	152	52	32.00%	BMI, fasting glucose, fasting insulin
Kang	2019	50-69	47.94%	1991	2967	1064	42.30%	BMI, TC, TG, HDL

The studies of van der Deure WM (a) and (b) represent the Rotterdam Study and the Rotterdam Scan Study, respectively. Age composition is expressed as mean ± standard deviation or age range. BMI, TC, TG, HDL, respectively, represent body mass index, total cholesterol, total triglyceride, and high-density lipoprotein cholesterol.

## Data Availability

The data analyzed in this study were all from the published articles. We have described the detailed retrieval process in Materials and Methods.

## Supplementary Materials

Supplementary Materials Supplementary Figure 1: pairwise comparison and standardized mean difference integration of the serum triglyceride levels of different genotypes. Supplementary Figure 2: pairwise comparison and standardized mean difference integration of the serum total cholesterol levels of different genotypes. Supplementary Figure 3: pairwise comparison and standardized mean difference integration of the serum high-density lipoprotein levels of different genotypes. Supplementary Figure 4: comparison of the hypertension prevalence of carriers and noncarriers.
