# Supplementary material for: The Type 2 Deiodinase Thr92Ala Polymorphism Is Associated with Higher Body Mass Index and Fasting Glucose Levels: A Systematic Review and Meta-Analysis
Source: Biomed Res Int. 2021 Oct 7;2021:9914009. doi: 10.1155/2021/9914009 (PMC8516525; doi:10.1155/2021/9914009)
Supplement: Supplementary Materials — Supplementary Figure 1: pairwise comparison and standardized mean difference integration of the serum triglyceride levels of different genotypes. Supplementary Figure 2: pairwise comparison and standardized mean difference integration of the serum total cholesterol levels of different genotypes. Supplementary Figure 3: pairwise comparison and standardized mean difference integration of the serum high-density lipoprotein levels of different genotypes. Supplementary Figure 4: comparison of the hypertension prevalence of carriers and noncarriers. [file 9914009.f1.docx]

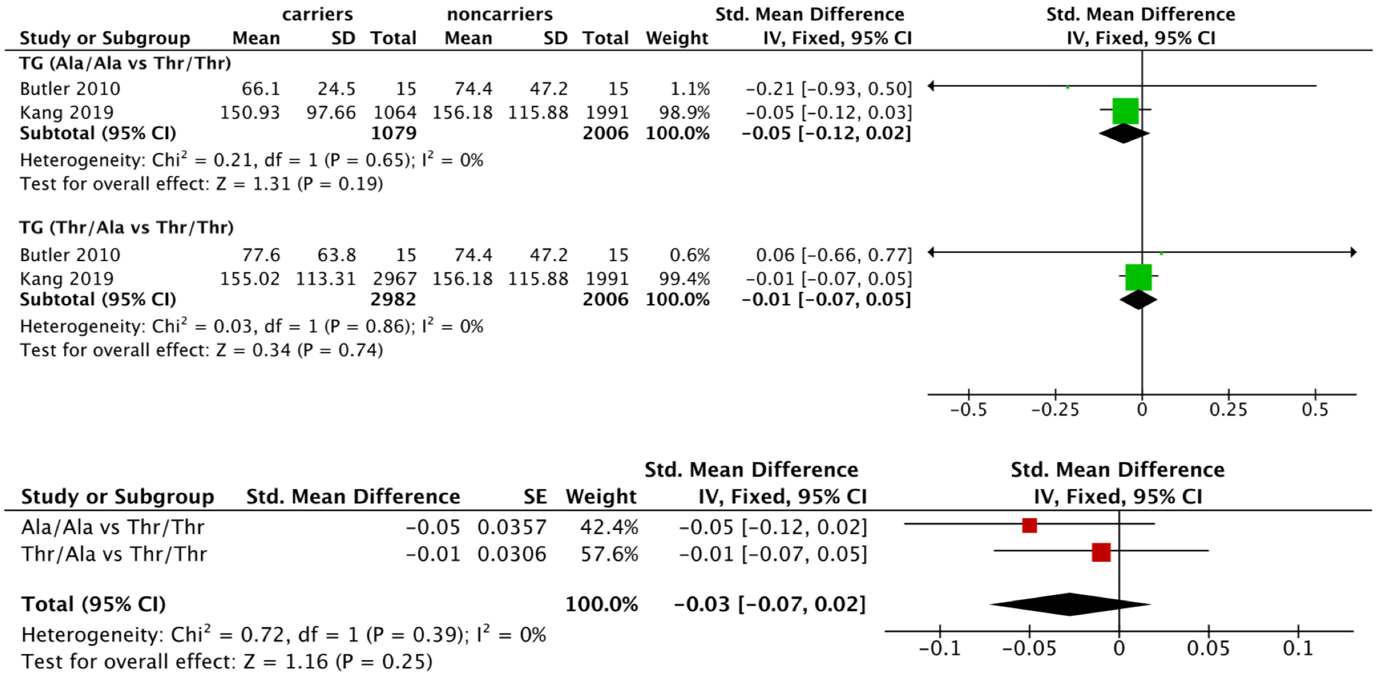


Supplementary Figure 1. Pairwise comparison and standardized mean difference integration of the serum triglyceride levels of different genotypes


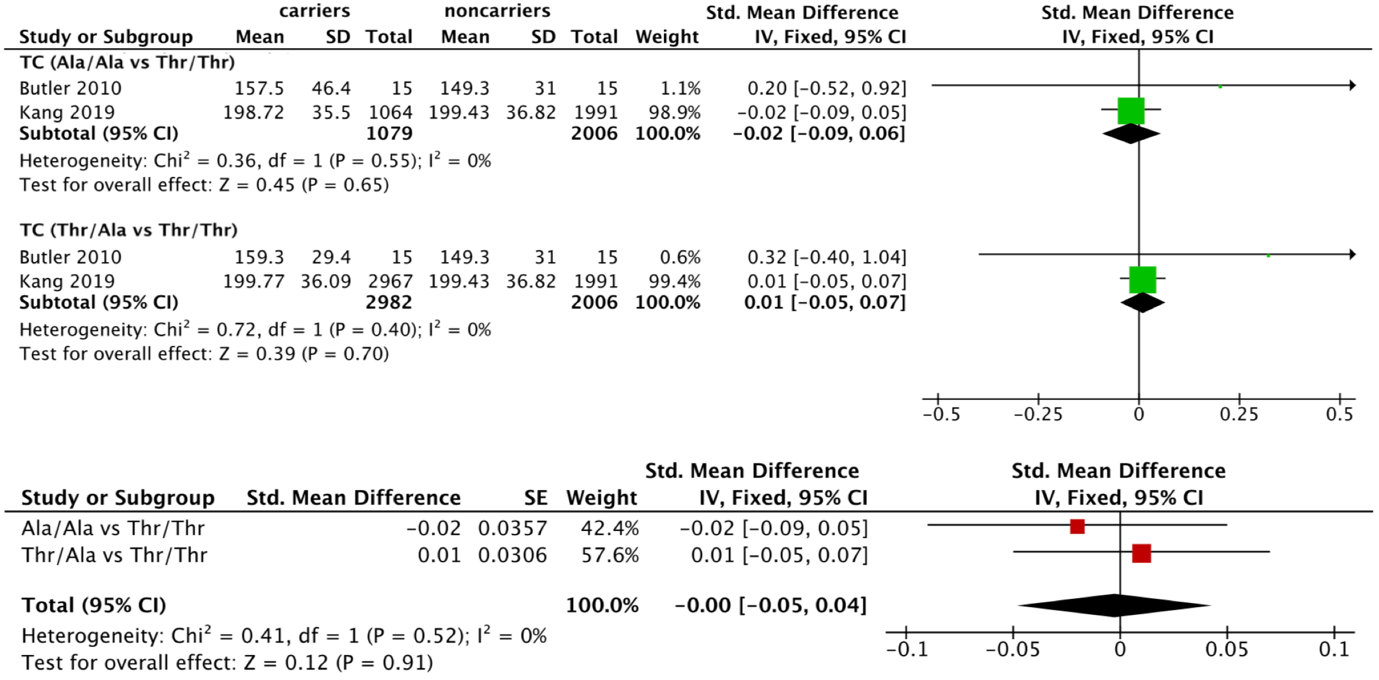


Supplementary Figure 2. Pairwise comparison and standardized mean difference integration of the serum total cholesterol levels of different genotypes


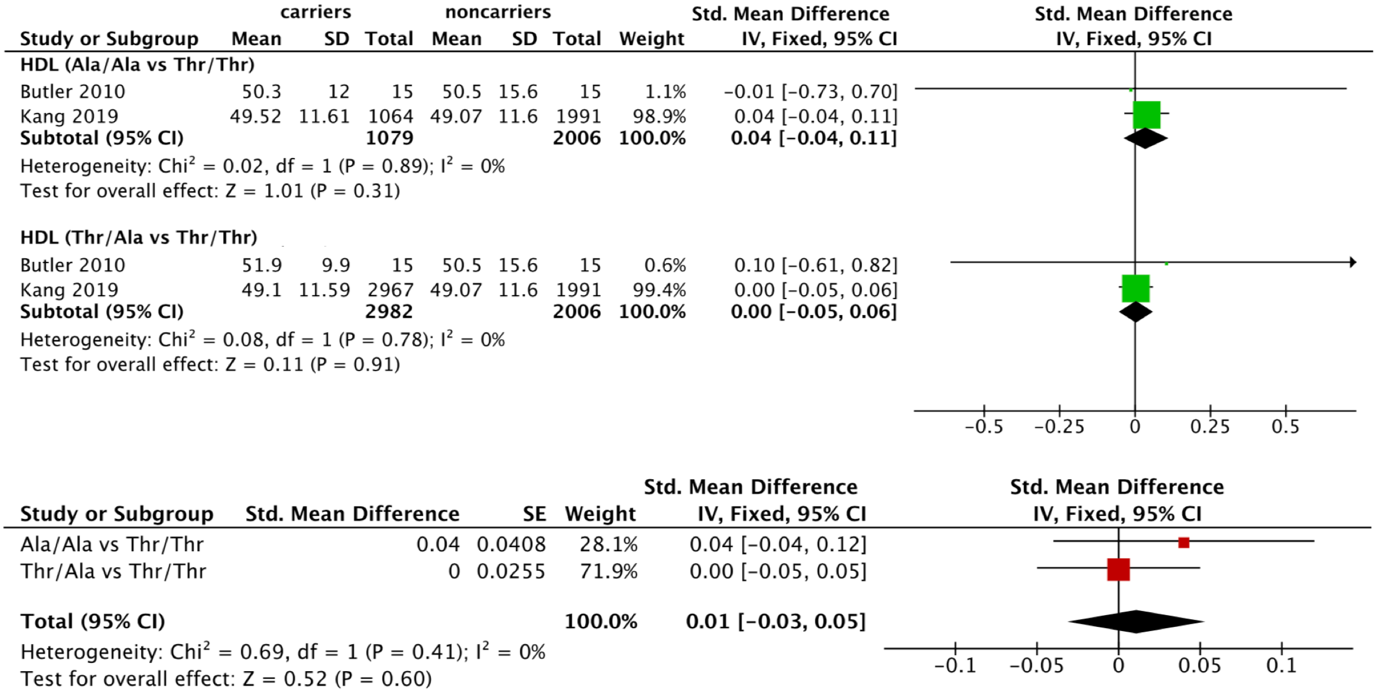


Supplementary Figure 3. Pairwise comparison and standardized mean difference integration of the serum high-density lipoprotein levels of different genotypes


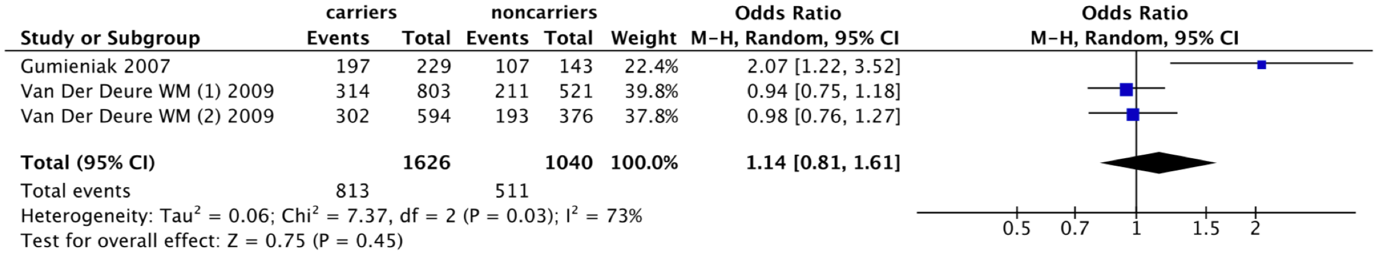


Supplementary Figure 4. Comparison of the hypertension prevalence of carriers and noncarriers. The studies of Var Der Deure WM (1) and (2) respectively represent the Rotterdam Study and the Rotterdam Scan Study.
